# Early Molecular Immune Responses of Turbot (*Scophthalmus maximus* L.) Following Infection with *Aeromonas salmonicida* subsp.* salmonicida*

**DOI:** 10.3390/ijms241612944

**Published:** 2023-08-18

**Authors:** Carlos Fajardo, Paulo Santos, Ricardo Passos, Mariana Vaz, Rita Azeredo, Marina Machado, Sergio Fernández-Boo, Teresa Baptista, Benjamin Costas

**Affiliations:** 1 Interdisciplinary Centre of Marine and Environmental Research (CIIMAR), University of Porto, 4450-208 Porto, Portugal; cquinones@ciimar.up.pt (C.F.); paulo.santos@ciimar.up.pt (P.S.); mleme@ciimar.up.pt (R.A.); mcasimiro@ciimar.up.pt (M.M.); sboo@ciimar.up.pt (S.F.-B.); 2 MARE—Marine and Environmental Sciences Centre, ESTM, Polytechnic Institute of Leiria, 2520-620 Peniche, Portugal; ricardo.passos@ipleiria.pt (R.P.); mariana.c.vaz@ipleiria.pt (M.V.); teresa.baptista@ipleiria.pt (T.B.); 3 Department of Biology, Faculty of Marine and Environmental Sciences, Instituto Universitario de Investigación Marina (INMAR), Campus de Excelencia Internacional del Mar (CEI·MAR), University of Cadiz (UCA), 11510 Puerto Real, Spain; 4 Department of Aquatic Production, School of Biomedicine and Biomedical Sciences, Abel Salazar Institute of Biomedical Sciences (ICBAS), University of Porto, 4050-313 Porto, Portugal

**Keywords:** furunculosis, gene expression, haematological profile, oxidative stress

## Abstract

Turbot aquaculture production is an important economic activity in several countries around the world; nonetheless, the incidence of diseases, such furunculosis, caused by the etiological agent *A. salmonicida* subsp.* salmonicida*, is responsible for important losses to this industry worldwide. Given this perspective, this study aimed to evaluate early immune responses in turbot (*S. maximus* L.) following infection with *A. salmonicida* subsp.* salmonicida*. For this, 72 fish were individually weighed and randomly distributed into 6 tanks in a circulating seawater system. For the bacterial challenge, half of the individuals (3 tanks with 36 individuals) were infected using a peritoneal injection with the bacterial suspension, while the other half of individuals were injected with PBS and kept as a control group. Several factors linked to the innate immune response were studied, including not only haematological (white blood cells, red blood cells, haematocrit, haemoglobin, mean corpuscular volume, mean cell haemoglobin, mean corpuscular haemoglobin concentration, neutrophils, monocytes, lymphocytes, thrombocytes) and oxidative stress parameters, but also the analyses of the expression of 13 key immune-related genes (*tnf-α*, *il-1β*, *il-8*, *pparα-1*, *acox1*, *tgf-β1*, *nf-kB p65*, *srebp-1*, *il-10*, *c3*, *cpt1a*, *pcna*, *il-22*). No significant differences were recorded in blood or innate humoral parameters (lysozyme, anti-protease, peroxidase) at the selected sampling points. There was neither any evidence of significant changes in the activity levels of the oxidative stress indicators (catalase, glutathione S-transferase, lipid peroxidation, superoxide dismutase). In contrast, pro-inflammatory (*tnf-α*, *il-1β*), anti-inflammatory (*il-10*), and innate immune-related genes (*c3*) were up-regulated, while another gene linked with the lipid metabolism (*acox1*) was down-regulated. The results showed new insights about early responses of turbot following infection with *A. salmonicida* subsp.* salmonicida*.

## 1. Introduction

Turbot (*Scophthalmus maximus* L.) (Linnaeus, 1758) aquaculture is an important economic activity in several countries of Europe (Spain, Portugal, France), Asia (China), and South America (Chile), of which Spain and Portugal are the most important producers [1,2,3]. Similar to other farmed fish species, turbot aquaculture is heavily affected by disease outbreaks. Among these, furunculosis, caused by *A. salmonicida* subsp.* salmonicida*, has produced important losses to the turbot industry [4,5]. *A. salmonicida* was reported for the first time in Germany in cultured brown trout (*Salmo trutta*) at the end of the nineteen century [6] and is currently one of the most important fish pathogens in aquaculture worldwide [7,8,9]. *A. salmonicida* affects mainly salmonid species, among which the Atlantic salmon (*Salmo salar*) is considered the most susceptible species [10,11,12]. Nonetheless, *A. salmonicida* has been shown to infect many other fish species, both in freshwater and marine environments [13].

Furunculosis, first described in turbot cultured in France and Spain in the early 1990s [14,15], is particularly critical in turbot production in Spain and Portugal [16]. Furthermore, epizootic outbreaks of acute furunculosis in turbot farms have been reported also in Denmark, Norway, and China, among other geographical regions [13,17,18]. A diagnosis of furunculosis in fish is generally made through pathogen isolation followed by serological or molecular identification techniques [19]. *A. salmonicida* is a non-motile Gram-negative bacterium that is ubiquitous in aquatic environments. Among its five subspecies (*salmonicida*, *achromogenes*, *smithia*, *pectinolytica*, and *masoucida*), *A. salmonicida* subsp.* salmonicida* is the most frequently detected in infected fish [20,21]. *A. salmonicida* is able to invade the host through injured skin and gills as well as the mucosa of the gastrointestinal tract [22,23]. An *A. salmonicida* systemic infection in turbot generally results in an acute fatal disease producing a hemorrhagic septicemia [24,25]. Differently, chronically infected specimens show multifocal granulomatous dermatitis [26], with single or multiple cutaneous nodules always situated on the ocular side of the body [27]. Cutaneous lesions linked to furunculosis include ulcers, erosions, and boil-like lesions known as furuncles, which have been described as raised vesicles containing a hemorrhagic fluid [28,29].

Previous reports about the description of immune responses of turbot after infection with *A. salmonicida* [5,24,26,27] highlight the presence of a response characterized not only by the over-expression of tumor necrosis factor-alpha (TNFα) protein produced by mononuclear phagocytes, but also for the generation of nitric oxide (NO) by the stimulation of the inducible nitric oxide synthase pathway (iNOS) [24]. Therefore, those are considered two of the main immune effectors used by turbot to eliminate *A. salmonicida* subsp. *salmonicida* [24]. Moreover, studies carried out by Coscelli et al. [26] describe immune reactivity against *A. salmonicida* antigens in both circulating and tissue monocytes/macrophages. These authors also highlights the phagocytosis as another key immune response during the initial phase of the infection [26]. Furthermore, histologic patterns studied by Coscelli et al. [27] indicate that the inflammation process begins in the deep dermis and, subsequently, extends towards the superficial dermis, generating ulceration and detachment of dermal tubercles in the most severe cases [27]. Additionally, Millan et al. [5] were able to identify 471 differentially expressed genes (DEGs) in turbot after infection with *A. salmonicida*: 246 in the liver, 223 in the spleen, and 125 in the head kidney, in at least 1 of the 5 sampled points (12 h, 1, 3, 7, and 21 days, respectively) for each organ [5].

The management of furunculosis by antibiotics is limited due to the fast development of bacterial resistance [30,31] and to their potential accumulation, both in the aquatic environment and/or in fish products [32,33]. Furthermore, there is a very limited number of antibiotics licensed in Europe for their use in aquaculture. Nonetheless, the control of the disease by the use of vaccines is still the most reasonable and environmental-friendly alternative. Different vaccines have been developed for the management of furunculosis [4,16,34] and adjuvants are an important tool to increase the efficiency of those vaccines [35], leading to a considerable reduction in the use of antibiotics [36]. However, the protection granted by these vaccines in turbot is low and lasts no more than 3–4 months [16,37]. Therefore, despite the efforts and methods deployed by the turbot aquaculture industry to deal with the negative effects caused by furunculosis, such as the use of antibiotics, vaccines, and adjuvants, the incidence of *A. salmonicida* is still prevalent and a major threat for the turbot aquaculture sector [24,26]. Given this perspective, the aim of this work was to reproduce experimentally the acute form of *A. salmonicida* subsp.* salmonicida* infection in turbot in order to obtain a more complete description of the immune response represented by a succession of phases of expression modulation of key immune-related genes.

## 2. Results

### 2.1. Haematological Analyses and Leucocytes Count

There were no significant differences in haematological parameters among infected and PBS groups. White blood cells (WBC), red blood cells (RBC), haemoglobin, mean corpuscular volume (MVC), and mean cell haemoglobin (MCH) parameters remained stable throughout the experiment. Similarly, the count of neutrophils, monocytes, lymphocytes, and thrombocytes did not present significant variations as a consequence of the infection. Nonetheless, in the case of haematocrit, a decreased tendency was evidenced at 9 h post infection, remained constant during 24 and 48 h post infection, respectively. In contrast, the mean corpuscular haemoglobin concentration (MCHC) registered a slight increased tendency at 48 h post infection. The complete set of results is available in Table S1.

### 2.2. Innate Humoral Parameters

No significant differences were recorded in any of the measured innate humoral parameters. The lysozyme (LYS), anti-protease (AP), and peroxidase (PER) activities remained stable during the experiment, not showing any important variation; however, in the case of PER activity, a certain downward trend is shown, reaching its peak at 24 h post infection. The complete set of results is available in the Table S2.

### 2.3. Oxidative Stress

No significant differences were found in the parameters of oxidative stress. Catalase (CAT), glutathione S-transferase (GST), lipid peroxidation (LPO), and superoxide dismutase (SOD) activities also remained without significant variations during the course of the challenge; however, in the case of the CAT, there was a slight downward trend that reaches its maximum point 9 h post infection. In contrast, GST and SOD activities show a slight upward trend, peaking at 48 h post infection. The complete set of results is available in the Table S2.

### 2.4. Gene Expression Analysis

The comparative statistical analysis revealed that only 5 of the 13 genes studied presented significant differences in their expression level comparing the infected groups with the respective PBS controls. In the case of the genes *tnf-α*, *il-1β*, *il-10*, *c3*, and *acox1*, significant differences were detected and corroborated between infected and PBS groups. 

The *tnf-α* gene was upregulated at 3 h post infection, but the expression rate returned to basal at 6 h. On the other hand, *il-1β* expression levels were higher in infected fish sampled at 3 and 6 h post infection than levels of control (PBS). The anti-inflammatory cytokine *il-10* was upregulated by infection at 6 and 9 h, but its expression levels at the remaining sampling points were similar to those before infection. Following an initial downregulation of *c3*, the expression was significantly enhanced at 9 h in infected fish compared to the control (PBS), returning to basal levels at 24 and 48 h.

In the case of *acox1*, this gene was significantly down-regulated relative to the control (PBS) in infected fish sampled at 9 h, returning to basal levels at 24 and 48 h. Finally, the expression of *il-22* did not show any significant difference between the different bio-groups.

Importantly, in the case of the genes *il-8*, *pparα-1*, *tgf-β1*, *nf-kB p65*, *srebp-1*, *cpt1a*, and *pcna*, although there are differences in the level of expression compared to the control group at 0 h, those differences are not corroborated by the respective internal control (PBS). Therefore, such differences could not be attributed entirely to the effects of the infection and is possible that stress produced in the fish is affecting in this regard, due manipulation and/or by the injection itself. The complete set of results is available in Table 1 and can be visualized through a heat map (Figure S1).

## 3. Discussion

Interestingly, during the course of the experiment no significant differences were detected in any of the haematological parameters studied, comparing between infected and control (PBS) groups. These results contrast with the observations recorded by Coscelli et al. [27], which indicate that, in the case of turbot infected with *A. salmonicida* subsp. *salmonicida*, the leukocyte population was increased and composed mainly of macrophages and lymphocytes, suggesting a cellular immune response linked to a hyper-sensitivity reaction [27]. Indeed, the absence of a more marked immune response, in terms of a significant variation on haematological parameters, innate humoral and oxidative activity, could be attributed to the fact that the strain used in this case (*A. salmonicida* subsp. *salmonicida*, IA.21.2) might not present, or has lost, the virulence factors characteristic of strains whose deleterious effects are more conspicuous [21,25,38].

Recent genomic studies carried out by Vasquez et al. [21] indicate that the differences found between typical and atypical strains of *A. salmonicida* are strictly related to the activity of inserted sequences. Therefore, the presence or absence of non-coding RNAs, transcription regulators, and especially virulence factors [25,38], constitute the most important differences between strains considered typical and atypical, also affecting their phenotype. Therefore, plasmidome profoundly affects not only the plasticity of the *A. salmonicida* genome but also its virulence. Strains considered typical harbor numerous plasmids that contain virulence factors that stimulate the generation of acute infections [21]. On the other hand, atypical strains only have a single large plasmid, which contains fewer genes encoding virulence factors, which is reflected in much less acute virulence and in the development of chronic infections [21]. In fact, a comparative genomic analysis revealed that the strains considered atypical belong to the subsp. *salmonicida* showing 99.55% ± 0.25% identity among themselves, which are also closely related to typical strains [21].

Nonetheless, the course of an inflammatory reaction is dependent on several variables: from the detection and interpretation of inflammatory signals that lead to the activation of the immune cells response, such as cytokines release, to the reaction of the surrounding tissues in response to the inflammatory triggers and the immune mediators [39]. Cytokines are cell-signaling proteins involved in haematopoiesis, inflammation, and defense against pathogens [40]. IL-1β can induce a cascade of reactions by activating leucocytes and stimulating the release of other cytokines [41], while TNF-α, a pleiotropic pro-inflammatory cytokine produced by numerous immune cells during inflammation [42,43], is involved in the process of cell apoptosis, proliferation, and differentiation, as well as in the regulation of other cytokines [44,45,46]. Given their potential to enhance and extend the inflammatory response, IL-1β and TNF-α are classified as pro-inflammatory cytokines, while others, such as IL-10 and TGF-β1, are regulatory anti-inflammatory mediators [47,48].

Results from the present study showed *il-1β* and *tnf-α* expression peaks at 3 and 6 h post infection followed by a gradual decrease in the later sampling point. Such expression patterns allow us to corroborate that these pro-inflammatory cytokines are key players during the first phase of the inflammatory response of turbot against *A. salmonicida* subsp. *salmonicida*. This pattern could be related to the expression levels of the anti-inflammatory cytokine IL-10, which reaches its peak of overexpression at 6 and 9 h post infection, revealing that it could be acting as a negative regulation mechanism of the inflammatory response in a second modulation phase. Indeed, IL-10 can induce immunosuppressive functions in several cytokines [49,50], including blocking TNF-α and IL-1β. In the case of *tgf-β1*, expression levels remained low throughout the experiment, a finding in line with that described previously for rainbow trout (*Oncorhynchus mykiss*). A down-regulation was observed for TGF-β1 transcripts at the early stages of viral infection with a highly virulent isolate of the viral hemorrhagic septicemia virus (VHSV), perhaps to allow a rapid inflammatory response to develop [51].

Furthermore, it has been demonstrated that diverse regulatory mechanisms of the inflammatory reaction are influenced by lipid and fatty acid-derived molecules. It has been proposed that PPARs can control the expression of genes related with the inflammatory responses indirectly and/or directly by trans-repression [39]. Moreover, it has been hypothesized that PPARs can modulate inflammatory responses indirectly through the alteration of the lipid metabolism; therefore, PPARs would modify both internal and extracellular content of lipid molecules, and this alteration of the lipid environment would trigger a secondary regulatory cascade of events [39]. For example, in the case of *acox1*, a key gene related with the hepatic metabolism, which is crucial for fatty acid oxidation [52,53,54], the level of expression decreases significantly at 9 h post infection. This transcript profile could be interpreted as an attempt by the host to modify the metabolism of lipids that are precursors of immune mediators, such eicosanoids and prostaglandins, in order to modulate the inflammatory response [39].

On the other hand, there were no significant changes in *il-22* expression level between the different experimental groups. IL-22 is a multifunctional cytokine (pro- and anti-inflammatory) that is mainly produced by T cells and natural killer cells [55]. The absence of a significant response from *il-22* in terms of its level of regulation could be interpreted as a lack of activation of T cells. For instance, TGF-β1 has dual key roles in Treg and Th17 cell differentiation by inducing IL-17 secretion in the presence of IL-6, while in the presence of IL-22, it has been shown to maintain immune tolerance by inducing Treg cells [56,57].

Oxidative stress is closely related to an inflammatory response since unbalance of redox homeostasis can cause cellular damage, thus activating the innate immune response or causing apoptosis [58]. The production of reactive oxygen species (ROS) triggers a cascade of events that culminates in the translocation of the transcription factor *nf-κB* to the nucleus where it regulates the transcription of pro-inflammatory cytokines, like TNF-α, prompting the inflammation process [59,60,61]. In this case, the expression of the *nf-kB p65* gene showed no significant differences post injection, remaining low until 48 h post infection. The expression of *nf-kB p65* is controlled by numerous factors, including cytokines, PAMPs, or even toxins from bacteria that can affect the expression of this NF-KB subunit. Moreover, this behavior could be related to the absence of a marked oxidative stress level. Indeed, no significant differences were recorded in relation to the activity of the oxidative stress parameters. Additionally, it could also be inferred that, in this particular case, the induction of pro-inflammatory cytokines might not be entirely mediated by the *nf-κB* pathway; instead, *tnf-α* would play a most important role in this regard [24,42].

In the present study, after a first phase of down-regulation (between 3 and 6 h post infection), *c3* showed a significant increase in its expression level at 9 h post infection, returning to its basal levels at 24 h post infection. The complement pathway is a biochemical cascade related with more than 35 soluble plasma proteins. This defense mechanism plays a key role linked to innate immunity, but it also shows the capacity to stimulate the specific immune response [62]. In the case of vertebrates, *c3* is proteolytically activated through a C3 convertase mediated by the lectins and/or the alternative complement pathways [63]. Indeed, turbot transcriptome shows many types of lectins, mostly C-type lectins [43], which are a family of carbohydrate-recognition proteins implicated in the first-line of defense against pathogens and immune regulation [64].

## 4. Materials and Methods

### 4.1. Experimental Design

Turbot juveniles were obtained from a local fish farm (ACUINOVA, Mira, Portugal) and transferred to the CIIMAR facilities (Matosinhos, Portugal). Fish were quarantined for a period of 30 days and fed twice a day (2% of the body weight). Then, 72 fish (19.71 ± 4.37 g) were individually weighed and randomly distributed into 6 tanks (100 L) of a recirculating seawater system (*n* = 12, density = 2.4 Kg/m^3^, photoperiod 12 h light/12 h dark). Physicochemical parameters, like pH (8.24 ± 0.08), salinity (36.33 ± 1.15), and oxygen saturation (8.22 ± 0.15 mg/L), were controlled on a daily basis. Ammonium/nitrite and temperature were kept constant throughout the entire experiment (NH_4_ and NO_2_; 0.17 ± 0.15 and 0.79 ± 0.33 mg/L, respectively; temperature 18.01 ± 0.30 °C).

### 4.2. Bacterial Growth and Inoculum Preparation

*A. salmonicida* subsp. *salmonicida* (strain IA.21.2) isolated from turbot was kindly provided by Prof. Alicia Toranzo (University of Santiago de Compostela, Spain). Bacteria were grown in plates with tryptic soy agar (TSA, 1.5% of NaCl) (Difco, Franklin Lakes, NJ, USA) for 24 h. Then, isolated colonies (3) were shifted to Erlenmeyer flasks containing 50 mL of tryptic soy broth (TSB, 1.5% of NaCl) (Difco) and cultured with continuous agitation for 18 h at 15 °C. According to a pre-challenge (LD_50_), bacterial concentration was measured at 600 nm and adjusted to 6.33 × 10^8^ CFU/mL.

### 4.3. Bacterial Challenge

After 2 weeks of acclimatization to the experimental conditions, half of the individuals (3 tanks with 36 individuals) were infected using a peritoneal injection with 100 µL of the aforementioned suspension (6.33 × 10^7^ CFU/fish), while the other half of individuals were injected with the same volume of PBS (1×, Gibco, New York, NY, USA) and kept as a control group.

### 4.4. Sampling

Both control and infected groups were sampled before infection (0 h), and then 3, 6, 9, 24, and 48 h after the bacterial challenge. Two fish per tank were randomly sampled at each time point (*n* = 6 per treatment, euthanized with 2-phenoxyethanol, 0.5 mL/L). Blood samples were collected from the caudal vessels using 1 mL syringes (previously prepared with 3000 U/mL of heparin). Blood samples were then placed in 1.5 mL heparinized tubes and gently homogenized for haematological analysis as described below. The remaining blood was centrifuged for 10 min at 10,000× *g* at 4 °C, and afterwards, plasma was collected and stored at −80 °C. The liver and head kidney were aseptically extracted for oxidative stress and gene expression analyses, respectively. Head kidney was stored in RNA later (1/10 *w*/*v*) at 4 °C for 24 h and then stored at −80 °C. The liver was immediately frozen using liquid nitrogen and stored at −80 °C. This study was carried out under the guidelines of the Portuguese Veterinary Authority following FELASA category C recommendations and accordingly to the normative of protection of animals used for scientific purposes (European Union directive 2010/63/EU).

### 4.5. Haematological Analyses and Leucocyte Counting

Before centrifugation of homogenized blood, a small aliquot was reaped for total white blood cell (WBC) and red blood cell (RBC) counts, haematocrit, and haemoglobin determination (SPINREACT, Spain). Mean corpuscular volume (MCV), mean corpuscular haemoglobin (MCH), and mean corpuscular haemoglobin concentration (MHCH) were calculated according to the following formulation:

- $\mathrm{MCV}\;\left(µm3\right)=\left(\frac{Ht}{RBC}\right)\times \;10$
- $\mathrm{MCH}\;\left(\frac{pg}{cell}\right)=\left(\frac{Hb}{RBC}\right)\times \;10$
- $\mathrm{MHCH}\;\left(\frac{g}{100\;mL}\right)=\left(\frac{Hb}{Ht}\right)\times \;100$

Total WBC (1/20 in heparinized Hank’s Balanced Salt Solution or HBSS) and RBC (1/200 in heparinized HBSS) counts were performed using a Neubauer chamber. Smears obtained from heparinized blood were spread from a single blood droplet and air-dried. After drying, the slides were fixed with a solution of formaldehyde and ethanol (90% absolute ethanol to 10% of 37% formaldehyde) for 1 min. Neutrophils were labelled through the detection of peroxidase activity revealed using the Antonow’s technique described by Afonso et al. [65]. Slides were stained with Wright’s stain (Haemacolor, Merck, Germany) and visualized under oil immersion (1000×). Leucocytes were identified and a differential count of neutrophils, monocytes, lymphocytes, and thrombocytes was conducted in a total of 200 cells/smear [66]. Relative counts were later translated into absolute values (×10^4^/mL) of each cell type using total WBC counts.

### 4.6. Innate Humoral Parameters

#### 4.6.1. Peroxidase (PER)

Total PER activity in plasma was measured following the procedure described by Quade and Roth [67]. The color change reaction and the optical density (OD) was read at 450 nm in a Synergy HT microplate reader (Biotek, Winooski, VT, USA). Two wells containing 150 µL of HBSS instead of plasma were used as blanks. PER activity (U/mL plasma) was determined defining 1 U of PER as that which produces an absorbance change of 1 OD.

#### 4.6.2. Anti-Protease (AP)

AP activity was measured following the method described by Machado et al. [68]. Phosphate-buffered saline alone was used as a blank solution in the protocol, and a reference sample was obtained using phosphate-buffered saline instead of plasma. The percentage of trypsin activity was calculated as follows:

- $\%\;\mathrm{non}-\mathrm{inhibited}\;\mathrm{trypsin}\;=\frac{\left(\mathrm{Sample}\;\mathrm{absorbance}\;\times 100\right)}{\mathrm{Reference}\;\mathrm{sample}}$
- % inhibited trypsin =100− % non−inhibited trypsin

#### 4.6.3. Lysozyme (LYS)

A turbidimetric assay was used to evaluate LYS activity following the method described by Costas et al. [69], with some modification. Briefly, a solution of *Micrococcus lysodeikticus* was prepared (0.25 mg/mL in 50 mM Na_2_HPO_4_ buffer, pH 6.2). Plasma samples (10 μL) were added in duplicates to 150 μL of the above-mentioned suspension in a microplate. To serve as controls, 160 µL of Na_2_HPO_4_ buffer and 160 µL of bacterial suspension were also added in duplicates. The reaction was carried out at 25 °C, and the absorbance (450 nm) was measured after 0.5 and 20 min. A standard curve was prepared based on a serially diluted, lyophilized hen egg white lysozyme (Sigma, Saint Louis, MO, USA) in Na_2_HPO_4_ buffer (50 mM Na_2_HPO_4_ buffer, pH 6.2). The amount of LYS in samples was calculated using the formula of the standard curve.

### 4.7. Oxidative Stress

#### 4.7.1. Liver Homogenization

Liver tissues were homogenized with 10 volumes of ultrapure water, using a pellet mixer. A 200 µL aliquot was transferred into a microtube with 4 µL of 2,6-Di-tert-butyl-4-methylphenol (BHT, 4% in methanol) for lipid peroxidation (LPO) evaluation. For the remaining analyses, 1 volume of tissue homogenate was mixed with 1 volume of potassium phosphate buffer (0.2 M, pH 7.4) and centrifuged at 10,000× *g* (4 °C) for 20 min to obtain the post mitochondrial supernatant fraction (PMS). The supernatants were stored at −80 °C.

#### 4.7.2. Protein Concentration

The total protein concentration of liver homogenates was measured using Pierce™ BCA Protein Assay kit (Thermo Scientific, Rockford, IL, USA), with bovine serum albumin (BCA) as the standard, according to the manufacturer’s instructions, and as described by Costas et al. [70]

#### 4.7.3. Lipid Peroxidation (LPO)

LPO activity was determined following the method described by Bird and Draper [71]. In brief, 100 μL of cold 100% trichloroacetic acid (TCA) was added to each sample and thoroughly mixed. Then, 1 mL of 0.73% 2-thiobarbituric acid (TBA), Tris-HCl (60 mM), and 0.1 mM DTPA (pH 7.4) solution was added to each sample and blanks (200 μL ultrapure water + 4 μL of BHT 4% in methanol + 100 μL of TCA + 1 mL of TBA). The mixtures were incubated for 1 h at 100 °C in a laboratory oven and then centrifuged for 5 min at 15,000× *g*. Finally, 200 µL of supernatant was transferred to a microplate in triplicates. The absorbance was measured at 535 nm, and LPO was expressed as nmol of thiobarbituric acid reactive substances (TBARS) formed per g of wet tissue.

#### 4.7.4. Catalase (CAT)

CAT activity was determined in PMS by measuring the substrate (H_2_O_2_) consumption, according to Claiborne [72], and adapted to a microplate [66]. Briefly, in a microplate well, 140 µL of phosphate buffer (50 mM pH 7.0) and 150 µL H_2_O_2_ solution (30 mM in phosphate buffer 50 mM pH 7.0) were added to 10 µL of liver PMS (0.7 mg mL^−1^ total protein). The absorbance was measured at 240 nm for 2 min (1 read every 15 s) in a microplate reader (BioTek Synergy HT, Winooski, VT, USA), and catalase activity was expressed in U per mg of protein, using the H_2_O_2_ molar extinction coefficient at 240 nm (43.6 M/cm).

#### 4.7.5. Superoxide Dismutase (SOD)

SOD activity was measured according to Flohé and Otting [73] and adapted to a microplate according to Lima et al. [74]. SOD activity was monitored using the cytochrome C method, with xanthine/xanthine oxidase as the source of superoxide radicals. A reaction solution containing 50 mM potassium phosphate buffer with 1 mM Na-EDTA (pH 7.8), 0.7 mM xanthine, 0.03 mM cytochrome C, 0.1 mM Na-EDTA, and 0.03 U/mL xanthine oxidase was added to previously diluted samples (0.3 mg/mL total protein content) in triplicate wells in a microplate reader (BioTek Synergy HT, Winooski, VT, USA). The absorbance was read at 550 nm for 3 min in intervals of 20 s at 25° C [66]. The activity is reported in units of SOD per mg of protein. One unit of activity was defined as the amount of enzyme necessary to produce a 50% inhibition of the cytochrome C reduction rate.

#### 4.7.6. Glutathione-S-Transferase (GST)

GST activity was determined following the method described by Frasco and Guilhermino [75]. Briefly, 50 μL of each sample (previously diluted in K-phosphate buffer, 0.1 M pH 7.4; 0.7 mg/mL final protein concentration) was added to triplicate wells of a microplate. Then, 250 μL of a reaction solution (0.2 M potassium phosphate buffer, pH 6.5), 10 mM reduced glutathione (GSH), and 60 mM 1-chloro-2,4-dinitrobenzene (CDNB; Alpha Aesar, Haverhill, MA, USA) was added to each well, following the methodology of Habig et al. [76]. Absorbance was recorded at 340 nm, for 5 min (1 read every 20 s). GST activity was expressed as mU per mg of protein, using the molar extinction coefficient at 340 nm (9.6 × 10^6^ M/cm) [77].

### 4.8. Gene Expression Analysis

Extraction of RNA was performed using the total RNA isolation kit (NZYTech, Lisbon, Portugal) according with manufacturer’s instructions. RNA concentration and purity were analysed with spectrophotometry using a DeNovix DS-11 FX (Wilmington, NC, USA). RNA integrity was verified through a 2% agarose gel. cDNA was obtained using the first-strand cDNA synthesis kit (NZYTech, Lisbon, Portugal) and was carried out in a Veriti DX 96-well thermal cycler (Applied Biosystems, Waltham, MA, USA). Real-time quantitative PCR was performed in duplicate for each sample using a CFX384 Touch Real-Time PCR Detection System (Biorad, Hercules, CA, USA) using 4.4 μL of diluted cDNA (20 ng/µL) mixed with 5 μL of NZYSpeedy qPCR Green Master Mix (NZYTech, Lisbon, Portugal) and 0.3 μL (10 μM) of each specific primer to a final volume of 10 μL. Thirteen genes were selected and analysed in relation with their influence on the immune response. Primer efficiency was tested for each gene (Table 2). Cycling conditions were the same among the different genes, consisting of one cycle of 95 °C for 10 min, followed by 40 cycles of 2 steps of 95 °C for 15 s, and 60 °C for 1 min, with a final cycle at 95 °C for 1 min, followed by 35 s at 60 °C, and ending at 95 °C for 15 s. In order to normalize the data (normalized gene expression, ΔΔCq), we measured the expression level of two reference genes as a normalization factor. To calculate gene expression levels, target genes were normalized using an elongation factor-1 alpha (*ef-1α*) and ribosomal protein S4 (*rps4*) as housekeeping references. A heat map was generated through the Heatmapper web server using the previously described gene expression data, normalized to control (0 h) and using the Average Linkage clustering method and Pearson distance measurement method [78].

### 4.9. Statistical Analysis

Data are presented as mean ± standard deviation (SD) of each experimental group. Data were analysed for normality and homogeneity of variance and Log transformed before statistical treatment when needed. Data were analysed using one-way ANOVA followed by a post hoc Tukey HSD test between all combinations of different groups. The level of significance used was *p ≤* 0.05 for all statistical tests. The study and statistical analysis of gene expression was carried out using the Bio-Rad CFX Maestro 1.0 Version 4.0.2325.0418 (Bio-Rad, Hercules, CA, USA) software. 

## 5. Conclusions

No alterations on haematological, innate humoral, and oxidative stress variables were observed. At the transcriptional level, the expression of immune-related genes points to an increase in pro-inflammatory cytokines (*tnf-α*, *il-1β*), anti-inflammatory cytokines (*il-10*) and an innate immunity-related gene (*c3*), and a decrease in a key gene related with the lipid metabolism (*acox1*), in a succession of phases of modulation of the immune response of turbot infected with *A. salmonicida* subsp.* salmonicida*.

## Figures and Tables

**Table 1:** Immune genes’ expression in the head kidney of *S. maximus* (L.) i.p. injected with *A. salmonicida* subsp.* salmonicida* (INF) or a placebo (PBS) and sampled at 3, 6, 9, 24, or 48 h post injection. Relative normalized gene expression (ΔΔCq). Values are presented as means ± SD (*n* = 6). If the difference was significant, according to one-way ANOVA (*p ≤* 0.05), a HDS Tukey post hoc test was used to identify differences in the experimental conditions. Letters represent differences among bio-groups. Symbol (*) indicates differences between the PSB and INF groups. Elongation factor-1 alpha (*ef-1α*) and ribosomal protein S4 (*rps4*) were used as reference genes.

Gene	0 h		3 h	6 h	9 h	24 h	48 h	*p* Value
*tnf-α*	1.00 ± 0.95 ^b^	PBS	0.99 ± 0.73 *	0.84 ± 0.85	1.13 ± 1.01	1.16 ± 0.75	0.87 ± 0.47	
		INF	7.82 ± 9.17 ^a^	3.29 ± 3.06 ^b^	1.29 ± 0.79 ^b^	1.34 ± 0.58 ^b^	1.46 ± 0.66 ^b^	<0.001
*il-1β*	1.00 ± 0.86 ^c^	PBS	0.31 ± 0.16 *	0.38 ± 0.13 *	1.06 ± 1.18	0.48 ± 0.21	0.52 ± 0.33	
		INF	4.40 ± 4.96 ^ab^	5.99 ± 7.73 ^a^	3.04 ± 2.16 ^bc^	1.56 ± 0.94 ^c^	0.88 ± 0.19 ^c^	<0.001
*il-8*	1.00 ± 0.40 ^a^	PBS	0.68 ± 0.26	0.67 ± 0.26	0.84 ± 0.42	1.12 ± 0.32	0.85 ± 0.14	
		INF	0.741 ± 0.35 ^ab^	0.64 ± 0.20 ^b^	0.79 ± 0.27 ^ab^	0.89 ± 0.19 ^ab^	1.00 ± 0.25 ^ab^	<0.001
*pparα-1*	1.00 ± 0.55 ^a^	PBS	0.55 ± 0.23	0.58 ± 0.26	0.60 ± 0.41	0.66 ± 0.18	0.59 ± 0.33	
		INF	0.46 ± 0.22 ^b^	0.35 ± 0.14 ^b^	0.34 ± 0.20 ^b^	0.47 ± 0.19 ^b^	0.67 ± 0.35 ^ab^	<0.001
*acox1*	1.00 ± 0.42 ^a^	PBS	0.69 ± 0.27	0.81 ± 0.21	1.21 ± 0.48 *	0.80 ± 0.39	0.78 ± 0.21	
		INF	0.59 ± 0.27 ^b^	0.61 ± 0.22 ^b^	0.80 ± 0.25 ^ab^	0.53 ± 0.18 ^b^	0.55 ± 0.22 ^b^	<0.001
*tgf-β1*	1.00 ± 0.42 ^a^	PBS	0.69 ± 0.16	0.72 ± 0.18	0.75 ± 0.34	0.68 ± 0.19	0.67 ± 0.10	
		INF	0.69 ± 0.28 ^bc^	0.66 ± 0.17 ^bc^	0.89 ± 0.22 ^ab^	0.72 ± 0.13 ^bc^	0.61 ± 0.13 ^c^	<0.001
*nf-kB p65*	1.00 ± 0.36 ^a^	PBS	0.53 ± 0.12	0.62 ± 0.14	0.72 ± 0.21	0.67 ± 0.15	0.60 ± 0.17	
		INF	0.70 ± 0.23 ^b^	0.76 ± 0.29 ^ab^	0.79 ± 0.16 ^ab^	0.61 ± 0.15 ^b^	0.58 ± 0.21 ^b^	<0.001
*srebp-1*	1.00 ± 0.41 ^a^	PBS	0.66 ± 0.24	0.66 ± 0.17	0.73 ± 0.23	0.65 ± 0.24	0.52 ± 0.16	
		INF	0.66 ± 0.20 ^bc^	0.74 ± 0.23 ^abc^	0.84 ± 0.34 ^ab^	0.50 ± 0.12 ^c^	0.60 ± 0.20 ^bc^	<0.001
*il-10*	1.00 ± 0.52 ^b^	PBS	1.04 ± 0.93	0.54 ± 0.20 *	1.32 ± 0.84 *	0.74 ± 0.30	0.77 ± 0.28	
		INF	3.35 ± 2.42 ^b^	10.35 ± 13.69 ^a^	14.88 ± 10.15 ^a^	3.28 ± 1.57 ^b^	2.00 ± 0.94 ^b^	<0.001
*c3*	1.00 ± 0.99 ^bc^	PBS	0.69 ± 0.30	0.63 ± 0.32	0.75 ± 1.00 *	0.64 ± 0.63	1.30 ± 0.62	
		INF	0.67 ± 0.55 ^bc^	0.44 ± 0.31 ^c^	2.27 ± 1.50 ^a^	1.63 ± 1.09 ^ab^	1.29 ± 0.39 ^bc^	<0.001
*cpt1a*	1.00 ± 0.43 ^a^	PBS	1.16 ± 0.33	0.97 ± 0.28	0.80 ± 0.52	0.87 ± 0.26	0.98 ± 0.27	
		INF	1.00 ± 0.40 ^a^	0.75 ± 0.26 ^ab^	0.50 ± 0.15 ^b^	0.95 ± 0.36 ^a^	0.86 ± 0.27 ^ab^	<0.001
*pcna*	1.00 ± 0.76 ^ab^	PBS	0.75 ± 0.53	0.88 ± 0.39	0.98 ± 0.47	0.59 ± 0.31	0.66 ± 0.36	
		INF	1.30 ± 0.65 ^a^	0.59 ± 0.26 ^bc^	0.83 ± 0.57 ^abc^	0.69 ± 0.24 ^abc^	0.53 ± 0.27 ^c^	<0.001
*il-22*	1.00 ± 0.97	PBS	0.61 ± 0.85	0.26 ± 0.23	0.65 ± 0.68	0.61 ± 1.02	0.27 ± 0.27	
		INF	0.47 ± 0.40	0.57 ± 0.45	0.27 ± 0.16	0.25 ± 0.14	0.74 ± 0.63	0.017

**Table 2:** Immune-related genes analysed using real-time qPCR.

Gene	Acronym	Efficiency (%)	Amplicon Length (bp)	Reference	Accession nº	Primer Sequence (5′-3′)
Interleukin 1 Beta	*il-1β*	98	181	[46]	AJ295836	F: TACCTGTCGTGCCAACAGGAA R: TGATGTACCAGTTGGGGAA
Interleukin 10	*il-10*	100.5	88	[79]	XM_035632547.1	F: AGTGACGGAGGACGCCAAGG R: ACATCTGCTGACATCGGACTTGAG
Acyl-CoA Oxidase 1	*acox1*	103.9	240	[80]	KC189925	F: AGTCCTCGCCCAGCTTTACT R: GGCTTCACATAGGTTCCGTCT
Complement Factor 3	*c3*	86.3	131	[81]	XM_035636545.1	F: GGTACAACTTCAACAACAACAACAA R: AGCGTAGTACAGCGACACCATT
Carnitine Palmitoryl-Transferase 1 Isoform A	*cpt1a*	107.2	96	[80]	XM_035614266.1	F: ATGGGAAGAGTGGACTGAATG R: GCTGGAAGGCATCTGTGG
Nuclear Factor Kappa B p65	*nf-kB p65*	106.3	127	[82]	MF370855	F: ATGCCTTTGAGGACCTTTT R: GTGTTCTGGGATGCTGTGT
Tumor Necrosis Factor Alpha	*tnf-α*	110.2	424	[46]	FJ654645	F: CCCTTATCATTATGGCCCTT R: TCCGAGTACCGCCATATCCT
Peroxisome Proliferator Activated Receptor Alpha 1	*pparα-1*	98.1	116	[83]	XM_035641791.1	F: GCGTCCCTTCAGTGATAT R: CTCCACAGCAGATGATAG
Interleukin 22	*il-22*	106	127	[43]	XM_035612680.1	F: TTGTTTTGAACTCCTCTGTGTGT R: GGATGGTGTCACCGTGGAAA
Transforming Growth Factor Beta 1	*tgf-β1*	111.7	153	[84]	XM_035623668.1	F: TCGCTTCCCGTTTCATCACT R: CCATGCTTTGCTCATTCCCG
Interleukin 8	*il-8*	116.5	184	[85]	XM_035651350.1	F: GGAGCCCAGAGTGTCGTTA R: CCTGCACCATAGAAATCTCATT
Proliferating Cell Nuclear Antigen	*pcna*	98	148	[86]	EU711051.1	F: GGTGGACGAGTGCTGCTTC R: CTGCCCTGCGGTACTAACC
Sterol Regulatory Element-Binding Protein 1	*srebp-1*	114.3	136	[80]	XM_035615397.1	F: GCCATTGACTACATCCGTTAC R: CATCAGCCTGTCCATCTACTTC
Ribosomal Protein S4	*rps4*	99.7	143	[87]	XM_035608277.1	F: CAACATCTTCGTCATCGGCAAGG R: ATTGAACCAGCCTCAGTGTTTAGC
Elongation Factor-1 Alpha	*ef-1α*	100.1	226	[80]	AF467776.1	F: TCATTGGCCATGTCGACTCC R: ACGTAGTACTTGGCGGTCTC

## Data Availability

Not applicable.

## Supplementary Materials

The following supporting information can be downloaded at: https://www.mdpi.com/article/10.3390/ijms241612944/s1.
