# Supplementary material for: Early Molecular Immune Responses of Turbot (Scophthalmus maximus L.) Following Infection with Aeromonas salmonicida subsp. salmonicida
Source: Int J Mol Sci. 2023 Aug 18;24(16):12944. doi: 10.3390/ijms241612944 (PMC10454659; doi:10.3390/ijms241612944)
Supplement: Supplementary file 1 [file ijms-24-12944-s001.zip › Figure S1.pdf]

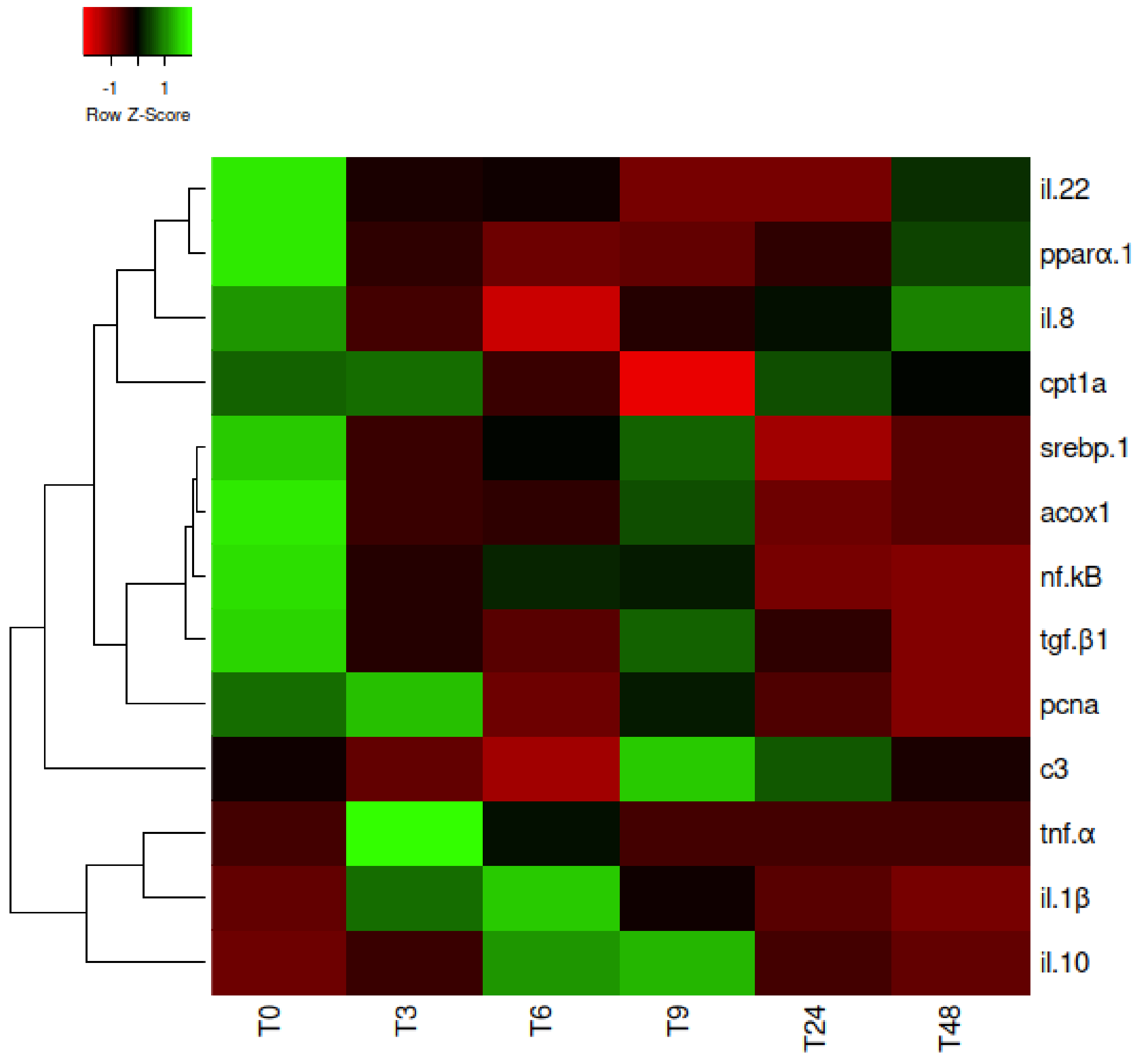

Figure S1. Heat map of gene expression in head-kidney of turbot infected with *A. salmonicida* subsp. *salmonicida*. Lines represent genes and columns represent different time points. (T0: control uninfected; T3: 3 h post infection; T6: 6 h post infection; T9: 9 h post infection; T24: 24 h post infection; T48: 48 h post infection). Different colors represent different levels of expression, ranging from low expression (red) to high expression (green).
