# Supplementary material for: Early Molecular Immune Responses of Turbot (Scophthalmus maximus L.) Following Infection with Aeromonas salmonicida subsp. salmonicida
Source: Int J Mol Sci. 2023 Aug 18;24(16):12944. doi: 10.3390/ijms241612944 (PMC10454659; doi:10.3390/ijms241612944)
Supplement: Supplementary file 1 [file ijms-24-12944-s001.zip › Table S1.pdf]

Table S1. Haematological parameters in *S. maximus* (L.) i.p. injected with *A. salmonicida* subsp. *salmonicida* (INF) or placebo (PBS) and sampled at 3, 6, 9, 24 or 48 h post injection. WBC (white blood cells, 10^4 cells / uL); RBC (red blood cells, 10^6 cells / uL); Haematocrit (%); Haemoglobin (g / dL); MCV (mean corpuscular volume, um^3); MCH (mean cell haemoglobin, pg / cell); MCHC (mean corpuscular haemoglobin concentration, g / 100 mL); Neutrophils (10^4 cells / uL); Monocytes (10^4 cells / uL); Lymphocytes (10^4 cells / uL); Thrombocytes (10^4 cells / uL). Values are presented as mean ± SD ($n = 6$). If the differences were significant, according to one-way ANOVA ($p \leq 0.05$), a HDS Tukey *post hoc* test was used to identify differences in the experimental conditions. Letters represent differences among bio-groups.

**Parameters**	**0 h**		**3 h**	**6 h**	**9 h**	**24 h**	**48 h**	***p* Value**
WBC	4.83 ± 1.51	PBS	3.98 ± 0.76	4.10 ± 1.41	4.55 ± 0.71	5.26 ± 1.75	3.68 ± 1.31	
		INF	4.23 ± 0.97	3.93 ± 1.75	4.48 ± 1.53	5.23 ± 1.50	4.15 ± 0.88	0.519
RBC	0.98 ± 0.16	PBS	1.07 ± 0.14	1.04 ± 0.12	0.92 ± 0.23	1.02 ± 0.17	0.96 ± 0.22	
		INF	0.95 ± 0.18	0.93 ± 0.20	1.12 ± 0.19	1.03 ± 0.09	1.01 ± 0.14	0.670
Haematocrit	17.67 ± 1.87 ^{a}	PBS	15.00 ± 3.35 ^{ab}	14.67 ± 1.97 ^{ab}	14.67 ± 1.75 ^{ab}	15.33 ± 2.06 ^{ab}	15.33 ± 1.75 ^{ab}	
		INF	15.00 ± 1.09 ^{ab}	14.17 ± 1.47 ^{ab}	13.50 ± 1.87 ^{b}	13.67 ± 1.63 ^{b}	13.83 ± 2.23 ^{b}	0.002
Haemoglobin	0.85 ± 0.24 ^{a}	PBS	1.03 ± 0.33 ^{a}	1.43 ± 0.70 ^{a}	0.95 ± 0.29 ^{a}	1.28 ± 0.32 ^{a}	2.60 ± 0.82 ^{b}	
		INF	1.29 ± 0.83 ^{a}	0.99 ± 0.30 ^{a}	1.14 ± 0.56 ^{a}	1.11 ± 0.24 ^{a}	1.62 ± 0.79 ^{ab}	< 0.001
MCV	171.90 ± 19.68	PBS	132.62 ± 22.00	141.77 ± 15.45	165.96 ± 32.22	142.44 ± 43.23	168.66 ± 47.60	
		INF	162.34 ± 29.75	156.94 ± 27.54	123.66 ± 22.91	133.33 ± 9.91	142.10 ± 45.97	0.06
MCH	8.23 ± 3.54 ^{a}	PBS	9.50 ± 2.20 ^{a}	13.80 ± 6.88 ^{ab}	10.51 ± 2.90 ^{a}	11.96 ± 5.01 ^{a}	22.11 ± 9.51 ^{b}	
		INF	13.13 ± 6.93 ^{ab}	10.82 ± 2.79 ^{a}	10.04 ± 3.82 ^{a}	10.95 ± 2.57 ^{a}	16.29 ± 6.98 ^{ab}	0.003
MCHC	4.40 ± 1.90 ^{a}	PBS	7.13 ± 0.92 ^{ab}	9.68 ± 4.44 ^{ab}	6.41 ± 1.48 ^{ab}	8.33 ± 1.69 ^{ab}	16.89 ± 5.30 ^{c}	
		INF	8.59 ± 5.50 ^{ab}	6.97 ± 1.85 ^{ab}	8.58 ± 4.61 ^{ab}	8.30 ± 2.14 ^{ab}	12.07 ± 6.29 ^{bc}	< 0.001
Neutrophils	1.21 ± 1.04	PBS	1.18 ± 0.68	1.25 ± 0.78	0.79 ± 0.34	1.07 ± 0.96	0.55 ± 0.29	
		INF	1.10 ± 0.42	0.71 ± 0.68	1.19 ± 0.91	1.67 ± 0.97	0.93 ± 0.43	0.203
Monocytes	0.04 ± 0.04	PBS	0.02 ± 0.02	0.06 ± 0.09	0.04 ± 0.04	0.02 ± 0.02	0.04 ± 0.05	
		INF	0.01 ± 0.01	0.02 ± 0.01	0.04 ± 0.07	0.03 ± 0.03	0.11 ± 0.07	0.163

Lymphocytes	0.80 ± 0.32	PBS	0.59 ± 0.35	0.85 ± 0.31	0.97 ± 0.26	1.49 ± 1.07	0.66 ± 0.28	
		INF	0.80 ± 0.18	1.07 ± 0.38	0.86 ± 0.34	0.97 ± 0.27	0.90 ± 0.11	0.054
Thrombocytes	2.77 ± 1.28	PBS	2.18 ± 0.76	1.93 ± 0.87	2.75 ± 0.70	3.27 ± 1.18	2.42 ± 1.14	
		INF	2.33 ± 0.80	2.14 ± 0.99	2.39 ± 0.53	2.56 ± 0.72	2.21 ± 0.79	0.475
