# Supplementary material for: Early Molecular Immune Responses of Turbot (Scophthalmus maximus L.) Following Infection with Aeromonas salmonicida subsp. salmonicida
Source: Int J Mol Sci. 2023 Aug 18;24(16):12944. doi: 10.3390/ijms241612944 (PMC10454659; doi:10.3390/ijms241612944)
Supplement: Supplementary file 1 [file ijms-24-12944-s001.zip › Table S2.pdf]

Table S2. Oxidative and innate humoral parameters in *S. maximus* (L.) i.p. injected with *A. salmonicida* subsp. *salmonicida* (INF) or placebo (PBS) and sampled at 3, 6, 9, 24 or 48 h post injection. CAT (catalase, U / mg); GST (glutathione S-transferase, mU / mg); LPO (lipid peroxidation, TBARS, nmol / g wt); SOD (superoxide dismutase, U / mg prot); LYS (lysozyme, µg / mL); AP (anti-protease, % Inhibited trypsin); PER (peroxidase; U / mL). Values are presented as means ± SD ($n = 6$). If the differences were significant, according to one-way ANOVA ($p \leq 0.05$), a HDS Tukey *post hoc* test was used to identify differences in the experimental conditions. Letters represent differences among bio-groups.

**Parameters**	**0 h**		**3 h**	**6 h**	**9 h**	**24 h**	**48 h**	***p* Value**
CAT	31.25 ± 12.83 ^{ab}	PBS	39.14 ± 13.67 ^{b}	22.67 ± 1.74 ^{ab}	23.22 ± 7.29 ^{ab}	20.64 ± 8.04 ^{a}	23.21 ± 13.86 ^{ab}	
		INF	25.62 ± 5.11 ^{ab}	23.74 ± 4.03 ^{ab}	18.47 ± 4.46 ^{a}	25.47 ± 4.96 ^{ab}	21.91 ± 6.40 ^{a}	0.009
GST	60.26 ± 12.50 ^{ab}	PBS	57.54 ± 10.23 ^{ab}	64.26 ± 20.71 ^{ab}	64.55 ± 13.67 ^{ab}	61.80 ± 6.49 ^{ab}	77.89 ± 12.10 ^{b}	
		INF	52.02 ± 11.08 ^{a}	53.62 ± 5.97 ^{a}	58.50 ± 6.65 ^{ab}	66.20 ± 8.00 ^{ab}	80.65 ± 18.60 ^{b}	0.002
LPO	24.62 ± 9.82 ^{ab}	PBS	24.03 ± 12.24 ^{ab}	18.77 ± 3,84 ^{ab}	17.34 ± 4.22 ^{ab}	26.48 ± 11.28 ^{b}	24.15 ± 2.54 ^{b}	
		INF	14.24 ± 2.67 ^{a}	16.78 ± 1.96 ^{ab}	18.90 ± 2.84 ^{ab}	24.89 ± 3.61 ^{b}	25.35 ± 5.55 ^{b}	0.001
SOD	90.01 ± 30.75 ^{a}	PBS	81.77 ± 20.17 ^{a}	75.58 ± 25.11 ^{a}	133.30 ± 54.23 ^{a}	64.81 ± 22.01 ^{a}	144.01 ± 84.33 ^{a}	
		INF	72.36 ± 34.70 ^{a}	104.40 ± 42.78 ^{a}	85.10 ± 40.85 ^{a}	106.05 ± 33.13 ^{a}	133.41 ± 40.60 ^{a}	0.007
LYS	2.44 ± 1.76	PBS	2.78 ± 3.28	3.33 ± 2.08	3.30 ± 2.52	2.93 ± 2.16	3.77 ± 1.02	
		INF	3.17 ± 1.12	2.28 ± 1.51	1.92 ± 0.69	3.71 ± 2.08	2.18 ± 2.41	0.874
AP	68.19 ± 8.31	PBS	68.62 ± 7.68	65.62 ± 13.90	75.52 ± 4.14	72.74 ± 13.90	66.94 ± 13.58	
		INF	70.28 ± 7.33	73.43 ± 8.50	60.70 ± 12.30	74.66 ± 5.67	74.39 ± 16.33	0.349
PER	79.43 ± 36.03	PBS	69.86 ± 30.63	45.66 ± 24.74	40.00 ± 21.64	32.82 ± 25.09	45.46 ± 27.42	
		INF	62.98 ± 39.67	63.67 ± 38.12	63.86 ± 43.56	29.95 ± 14.70	53.64 ± 34.60	0.071
